# The same chemical state of carbon gives rise to two peaks in X-ray photoelectron spectroscopy

**DOI:** 10.1038/s41598-021-90780-9

**Published:** 2021-05-27

**Authors:** G. Greczynski, L. Hultman

**Affiliations:** Thin Film Physics Division, Department of Physics, Chemistry, and Biology (IFM), Linköping University, 581 83 Linköping, Sweden

**Keywords:** Characterization and analytical techniques, Materials science, Surfaces, interfaces and thin films

## Abstract

Chemical state analysis in X-ray photoelectron spectroscopy (XPS) relies on assigning well-defined binding energy values to core level electrons originating from atoms in particular bonding configurations. Here, we present direct evidence for the violation of this paradigm. It is shown that the C 1s peak due to C–C/C–H bonded atoms from adventitious carbon (AdC) layers accumulating on Al and Au foils splits into two distinctly different contributions, as a result of vacuum level alignment at the AdC/foil interface. The phenomenon is observed while simultaneously recording the spectrum from two metal foils in electric contact with each other. This finding exposes fundamental problems with the reliability of reported XPS data as C 1s peak of AdC is routinely used for binding energy scale referencing. The use of adventitious carbon in XPS should thus be discontinued as it leads to nonsense results. Consequently, ISO and ASTM charge referencing guides need to be rewritten.

## Introduction

With more than 12,000 papers published annually, the value of XPS in materials science can hardly be overestimated^1^. The tremendous growth of the XPS technique is driven by the possibility of chemical state identification^2,3^, enabled nearly 60 years ago by the first observation of S 2p peak splitting in the XPS spectrum of sodium thiosulfate^4^, caused by the fact that S atoms in Na_2_S_2_O_3_ are present in two distinctly different chemical environments. However, significant fraction of these numerous XPS papers contains data that have been wrongly interpreted due to the lack of skills, experience, or knowledge^5^, but also because an improper referencing method was employed^6^.

The chemical state identification is conventionally done by comparing the extracted binding energy (BE) values to compound reference data bases such as the NIST XPS^7^. For the latter to be reliable, the spectrometer has to be correctly calibrated^8^. This does not, however, guarantee that the BE of energy level of the sample of interest for study (different from the calibration set) is correctly reproduced. The primary reason for this is the possibility of positive charge accumulation in the sample surface region (*surface charging*)^9^. The charge neutrality condition requires that the loss of negative charge from the surface region (the consequence of the photoelectric effect) is compensated with sufficiently high rate by electrons from the sample bulk, the substrate, or the surrounding environment. If that does not take place, the surface charges positively, which effectively lowers kinetic energy of emitted photoelectrons due to the Coulomb interaction and, in consequence, results in an uncontrolled shift of spectral peaks towards higher BE values.

To neutralize the negative charge loss and to enable spectra acquisition from poorly-conducting samples, low-energy electrons^10^ or a combination of electrons and ions (supplied by the so-called flood gun) are used. This, however, does not solve the referencing problem as one can never a priori assume that the surface is neutral during an XPS measurement. In order to distinguish peak shifts caused by charging from those due to chemistry, an internal reference level is necessary to establish.

The availability of an internal energy reference, in general, does not present a big challenge for conducting materials in electrical contact to the spectrometer. Such samples typically exhibit a clear cut-off in the density of states at the Fermi level (so-called Fermi edge, FE), which serves as a natural zero on the BE scale^11^.

The situation is, however, cumbersome for insulators, which dominate in XPS analyses. Here, by far the most common charge referencing method is the one that relies on the adventitious carbon (AdC) contamination that occurs on essentially all samples analyzed by XPS^3,12,13^—which accounts for the extreme popularity of this referencing technique. The method is temptingly simple and requires no other effort than recording the C 1s peak of AdC and setting the C–C/C–H component at the BE arbitrary chosen from the range 284.6 to 285.2 eV, as recommended by the ISO charge referencing guide^13^. The same rigid BE shift is then applied to all sample signals, hence assuming that the correction is independent of the electron kinetic energy.

Here, we present direct evidence, which should terminally disqualify the charge referencing method based on the C 1s peak of AdC, and refute the deep-rooted notion that the same chemical state gives rise to peaks at well-defined BE values.

## Results

**Figure 1:**
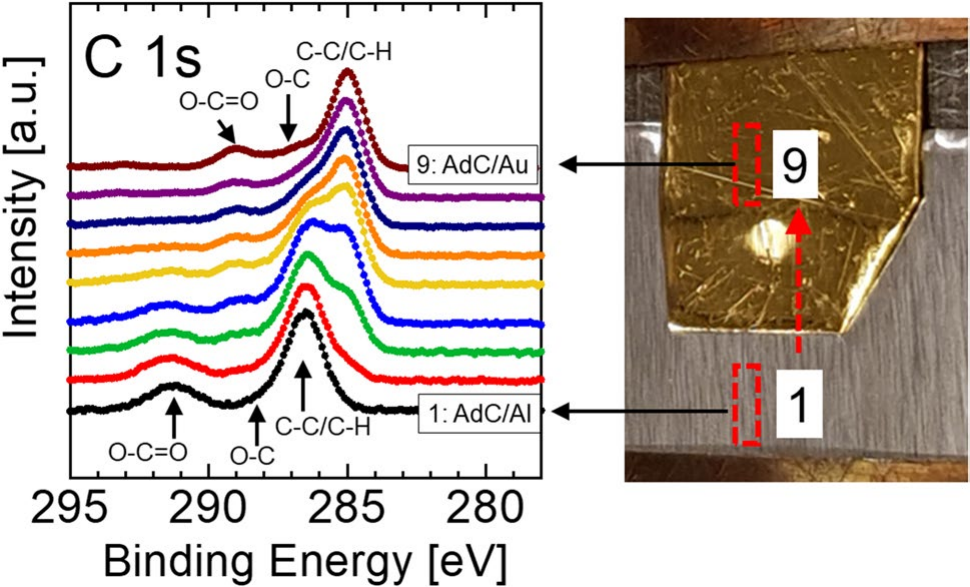
C 1s spectra of adventitious carbon recorded from Al and Au foil samples (showed to the right) as a function of lateral position, which varies from Al foil (1) to Au foil (9) in steps of 0.1 mm. The size of the area probed in each measurement is 0.3 × 0.7 mm^2^ (indicated with red rectangles).

Commonly available Al and Au foils with AdC layers resulting from prolonged air exposure are set in contact and mounted together on the sample holder. The AdC layer thickness estimated from the attenuation of the substrate signal using the electron mean free path values reported in Ref.^14^ are 2.1 and 3.2 nm for Al and Au substrates, respectively. The spectra are recorded from the area 0.3 × 0.7 mm^2^ that is sequentially moved from the Al part (position 1) to the Au part (position 9) with the step of 0.1 mm. As illustrated in Fig. 1, the binding energy of the C–C/C–H peak that dominates the C 1s spectra of AdC, with the envelope characteristic of adsorbed AdC, depends strongly on which sample area is probed and varies from 286.6 eV for the data recorded from the Al foil (bottom spectrum) to 285.0 eV for AdC/Au (top). Moreover, for the intermediate probe placements a double C–C/C–H peak is observed. Noteworthy, peaks due to two other chemical states of C atoms in AdC, O=C–O and C–O, shift in the same manner from higher to lower BE, as the probe is moved from Al to Au foil. This result contradicts the XPS paradigm, in which BE of core level peaks is defined by the type and the nature of chemical bonds.

**Figure 2:**
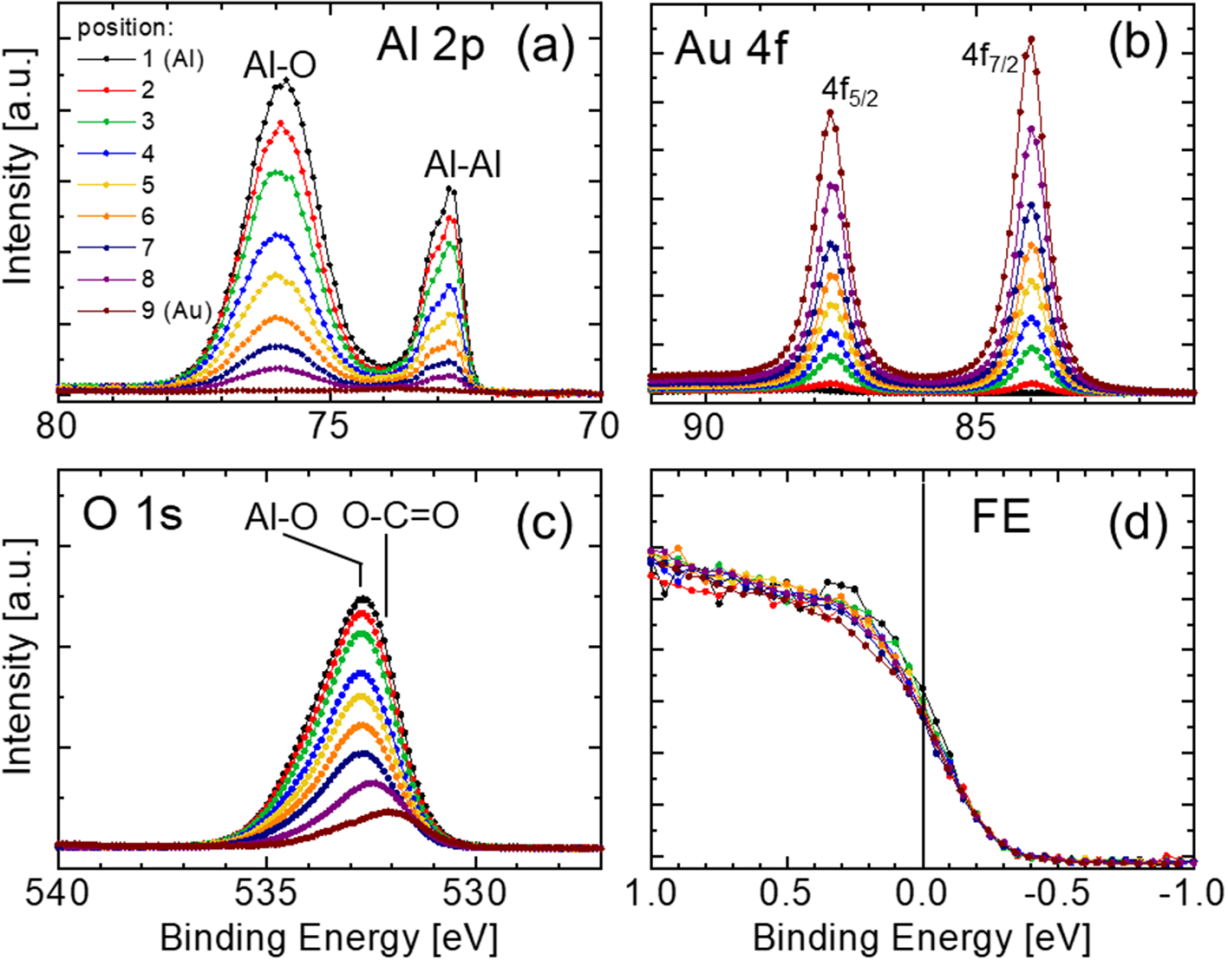
(**a**) Al 2p, (**b**) Au 4f, (**c**) O 1s, and (**d**) valence band spectra in the vicinity of the Fermi level recorded from Al and Au foil samples as a function of lateral position, which varies from Al foil (1) to Au foil (9) in steps of 0.1 mm. The size of the area probed in each measurement is 0.3 × 0.7 mm^2^.

To exclude possible influence of surface charging phenomena, Al 2p, Au 4f, and O 1s core level spectra are also recorded and shown in Fig. 2 together with the portions of the VB spectra in the vicinity of the Fermi level. Al 2p spectra consist of two contributions: metallic Al peak centered at 72.98 eV (with apparent asymmetry due to the unresolved 2p_3/2_-2p_1/2_ spin-splitting)^15^ and a broad Al–O peak at ~ 76 eV. The identity of the latter peak was verified in a separate experiment, in which evolution of Al 2p spectra was studied as a function of Ar^+^ sputter etch time (not shown). This test confirmed that after removal of surface oxides (evidenced by the loss of O 1s intensity) the higher BE peak disappeared, while the lower BE peak due to metallic Al increased in intensity and remained at the same binding energy. With moving from position 1 (Al foil) to position 9 (Au foil) the Al 2p signal intensity gradually decreases (with no change in the peak positions), while the intensity of Au 4f peaks (see Fig. 2b) increases. The Au 4f_7/2_ component is present at 84.0 eV, independent of probe placement. Thus, substrate signals are recorded at BE values typical for metallic Al and Au, which proves good electrical contact between specimens and spectrometer. The latter is further verified by recording portions of valence band spectra in the close vicinity of the Fermi level/edge. As depicted in Fig. 2d), the clear cut-off in the density of states coincides with the 0 eV on the BE scale, irrespective of which sample area is probed. These observations indicate that surface charging phenomena are not present.

Corresponding O 1s spectra in Fig. 2c are fully consistent with the evolution of Al 2p, Au 4f, and C 1s signals. A broad peak centered at 532.7 eV is observed in data obtained from Al foil, with unresolved contributions due to Al–O, C–O, and O–C=O (all contributions need to be present based on the corresponding Al 2p and C 1s spectra). By moving from Al to Au foil, the intensity of that broad peak decreases (predominantly due to the loss of the Al–O component) and eventually for the spectrum recorded at position 9 the entire signal originates from C–O and O–C=O bonds in the AdC layer present on the Au foil.

## Discussion

The all-important question is: -why does the binding energy of C 1s electrons originating from the C–C/C–H bonded carbon vary between Al and Au foils? This observation is highly disturbing as it speaks against the common belief that the chemical state determines BE of photoelectron peaks. Moreover, BE of the C 1s of AdC varying in such wide range presents serious problem for the validity of XPS reports that use this signal for referencing XPS spectra.

Such disturbing result is a direct consequence of the fact the AdC layer does not remain in proper electrical contact to the underlying substrate (likely due to fact that these carbonous species are physisorbed and can be easily removed by gentle heating)^16^ resulting in vacuum-level alignment at the AdC/sample interface^17^. Under these conditions, the measured BE values of all core level peaks from AdC layer (including of course C 1s) are decoupled from the spectrometer Fermi level and become a sensitive function of the sample work function ${\phi }_{SA}$. In an earlier paper, we established experimentally that the following relation holds: ${E}_{B}^{F}+{\phi }_{SA}$ = 289.58 ± 0.14 eV^18^, in which ${E}_{B}^{F}$ stands for the measured BE of the C 1s peak. The work function values for Al and Au foils, determined here from the secondary electron cut-off using the standard UPS procedure, are 3.0 and 4.5 eV, respectively, which accounts for the 1.5 eV difference in the BE of corresponding C 1s peaks of AdC (cf. Figure 1). Hence, independent of the substrate type, the BE of the C–C/C–H signal is essentially constant with respect to the vacuum level and amounts to 289.6 and 289.5 eV, for AdC layers on Al and Au foils, respectively.

The consequences of the above are disastrous for the use of the C 1s peak of AdC in referencing XPS spectra. First, the BE of this signal is determined by the specimen work function, which is a very sensitive property influenced by many variables like surface cleanness, roughness, crystalline phase, or crystal orientation. This practically disqualifies C 1s peak as reliable reference. Secondly, referencing the spectra to C 1s peak leads to unphysical results for specimens with relatively low work function (that is high BE of the C 1s line). For example, if the XPS spectra obtained from the Al foil were referenced by setting the C–C/C–H C 1s peak at the ISO/ASTM-recommended value of 284.8 eV, the metallic Al 2p would appear at 71.2 eV, which is ca. 1.8 eV lower BE than commonly accepted value for metallic Al. Moreover, the density of states would extend up to 1.8 eV above the Fermi level, which is obviously false.

Corresponding problems occur for any other arbitrary choice of the C 1s peak position within the ISO/ASTM recommended range (284.6–285.2 eV)^12,13^.  An additional complication is that AdC is an ill-defined compound in itself and may be changeable between experiments^19^.

The C 1s method has a history dating back to the early days of XPS, marked with rather extensive criticism expressed in the time period following the introduction in 1967 and stretching to 1982, when the critical review on the topic was published by Swift under the rhetoric title “*Adventitious Carbon-The Panacea for Energy Referencing?*”^20^. The objections concerned the unknown chemical composition of the AdC layer^21^ its unknown origin^22^, and the uncertain position of the C 1s peak^23,24^. Over time, sporadic critical voices^25–28^ became overrun by an avalanche of XPS papers that rely simply on the AdC referencing. Systematic studies on the use of AdC layers for BE referencing undertaken in our laboratory in the recent years fully confirm early objections to this technique and identified additional problems^17–19^.

Doubts over the C 1s method are to some extent reflected in the ISO 19,318:2004 document: “Surface chemical analysis—Reporting of methods used for charge control and charge correction” as well as in the ASTM E1523-15 standard “Charge Control and Charge Referencing Techniques in X-Ray Photoelectron Spectroscopy”. Although the method is recommended, careful reading reveals a certain reservation: “*A significant disadvantage of this method lies in the uncertainty of the true nature of the carbon and the appropriate reference values which, as reported in the literature, have a wide range from 284.6 eV to 285.2 eV for the C 1s electrons from hydrocarbon and graphitic carbon.*” Strikingly, in the same document it is stated that “*These contamination layers can be used for correction purposes ﻿* if it is assumed that they truly reflect the steady-state static charge exhibited by the specimen surface and that they contain an element with a peak of known binding energy. [our underlining]” That disclaimer basically leaves all the responsibility for using the C 1s method up to the investigator by putting him/her in a Catch-22 situation: the use of the referencing method is granted provided that it is possible to assess the sample charging state, for which one needs the concerned referencing method.

## Conclusions

In summary, we show that the C 1s signal from C–C/C–H bonded atoms present in adventitious carbon layers accumulating on Al and Au foils appears at two distinctly different binding energy values, thus violating the XPS paradigm of chemical state identification. As this signal is commonly used for referencing XPS spectra we argue that the ISO and ASTM charge referencing guides should be revoked. The here presented evidence for the failure of the conventional charge referencing procedure is based on readily available materials and methods and, as such, can be easily verified in any XPS laboratory. Since other alternatives such as noble metal decoration^29^, noble gas atom implantation^30^, deposition of organic layers^31^, “biased” referencing^32^, or the use of Auger parameter^33^ are not free from serious limitations, the lack of a reliable energy reference remains a fundamental problem in XPS analyses of insulating materials with far reaching consequences for many fields of modern materials science.

## Methods

Commonly available Al and Au foils are used in these experiments. Samples have been stored in the same laboratory air for the time period of at least one year before loading in the load lock chamber of an Axis Ultra DLD spectrometer from Kratos Analytical (UK) used for all XPS experiments here. The base pressure during spectra acquisition is lower than 1.1 × 10^–9^ Torr (1.5 × 10^–7^ Pa). The excitation source is monochromatic Al Kα radiation (hυ = 1486.6 eV) and the anode power is 150 W. All spectra are collected at normal emission angle. The analyzer pass energy is set to 20 eV, which results in that the spectrometer energy resolution determined from the FE cut-off of Au and Ag samples is 0.38 eV. The calibration of the binding energy scale was confirmed by examining sputter-cleaned Au, Ag, and Cu samples (all in the form of polycrystalline thin films) according to the recommended ISO standards for monochromatic Al Kα sources that place Au 4f_7/2_, Ag 3d_5/2_, and Cu 2p_3/2_ peaks at 83.96, 368.21, and 932.62 eV, respectively. For all measurements on sapphire, the charge neutralizer is used. Work function ${\phi }_{SA}$ measurements by ultraviolet photoelectron spectroscopy (UPS) are performed in the same instrument for films on Si(001) substrates with unmonochromatized He I radiation (hυ = 21.22 eV), immediately after XPS analyses, employing the standard procedure in which ${\phi }_{SA}$ is assessed from the secondary-electron cutoff energy in the He I UPS spectra^34^, with an accuracy of ± 0.05 eV.
